# Chromium in Postmortem Material

**DOI:** 10.1007/s12011-018-1328-8

**Published:** 2018-04-17

**Authors:** Danuta Dudek-Adamska, Teresa Lech, Tomasz Konopka, Paweł Kościelniak

**Affiliations:** 1 Department of Analytical Chemistry, Faculty of Chemistry of the Jagiellonian University, Gronostajowa 2, 30-387 Kraków, Poland; 2 Institute of Forensic Research, Westerplatte 9, 31-033 Kraków, Poland; 3 Department of Forensic Medicine, Jagiellonian University Medical College, Grzegórzecka 6, 31-531 Kraków, Poland

**Keywords:** Chromium concentration, Blood, Human organs, ETAAS

## Abstract

Recently, considerable attention has been paid to the negative effects caused by the presence and constant increase in concentration of heavy metals in the environment, as well as to the determination of their content in human biological samples. In this paper, the concentration of chromium in samples of blood and internal organs collected at autopsy from 21 female and 39 male non-occupationally exposed subjects is presented. Elemental analysis was carried out by an electrothermal atomic absorption spectrometer after microwave-assisted acid digestion. Reference ranges of chromium in the blood, brain, stomach, liver, kidneys, lungs, and heart (wet weight) in the population of Southern Poland were found to be 0.11–16.4 ng/mL, 4.7–136 ng/g, 6.1–76.4 ng/g, 11–506 ng/g, 2.9–298 ng/g, 13–798 ng/g, and 3.6–320 ng/g, respectively.

## Introduction

Chromium (Cr) is one of the heavy metals that is important for humans [1]. It is present in air, water, soil, and any living matter from natural and anthropogenic sources, with the largest release occurring from industrial (metallurgical, refractory, and chemical) sources.

The leading consumer of chromium materials, with ferrochromiums as the main components, is the stainless steel industry. In the refractory industry, chromium is mainly used in linings for high temperature industrial furnaces, while in the chemical industry, it is used primarily in pigments. Other applications include metal finishing, leather tanning, wood preservatives, catalysts, and miscellaneous applications, such as drilling mud, chemical manufacturing, textiles, toners for copying machines, magnetic tapes, and dietary supplements (for example chromium picolinate) [2, 3]. What is more, chromium alloys are also used in metal joint prostheses [2, 4, 5].

However, the general population is primarily exposed to this element by ingesting food [6]. Other routes of exposure like inhalation of ambient air, drinking water, or skin contact with certain consumer products or soils that contain chromium are of rather minor importance [2]. Chromium-rich food includes entrails, meat, mollusks, lobsters, vegetables, bran, whole wheat or rye bread, and unrefined sugar [1, 3].

The estimated safe daily dose of Cr(III) is 50 to 200 μg [3], while an adequate intake determined by the Institute of Medicine (IOM) of the National Research Council (NRC, USA) is 20–45 μg Cr(III)/day for adolescents and adults [2].

After ingestion, Cr(III) is absorbed mainly in the small intestine, then transported through blood to the cells and relatively quickly absorbed by bones, also accumulating in the spleen, liver, and kidneys [7]. The IOM reported average total plasma chromium concentrations of 0.10–0.16 μg/L and an average urinary chromium excretion of 0.22 μg/L or 0.2 μg/day [2].

Despite the growing interest in speciation analysis of different biologically important chromium forms—Cr(III), which is recognized as a trace element and Cr(VI), which is considered to be toxic, mutagenic, and carcinogenic—determination of its total content in available biological materials (such as body fluids and tissues) is still of great importance to clinical and forensic toxicology. This is because the knowledge of total content allows us to determine so-called “reference values/ranges” (for non-exposed and non-poisoned people), which can later be used for comparative purposes (confirmation or exclusion of any metal compound poisoning), for exposure assessment (e.g., environmental or occupational), as well as in the diagnosis of certain medical conditions. It is considered that chromium in the lungs is a result of deposition from inhaled air, whereas chromium in food is the main source in other organs [6]. For example, Antilla et al. [8] found that the average lung tissue concentration of total chromium in smokers was 6.4 μg/g versus 2.2 μg/g in non-smokers. Concentrations of total chromium in biological material in fatal cases of poisonings with chromium compounds are significantly higher (stomach 6–34 μg/g, liver 42–320 μg/g, kidneys 33–220 μg/g, blood 11 μg/mL, urine 18–101 μg/mL) [9].

However, current data on the concentration of total chromium in the human body in non-exposed people are mostly limited to concentrations found in whole blood [10–23], serum [11–13, 16, 24, 25], urine [11, 13–15, 17, 19, 20, 22, 25–34], or hair [17, 19, 20, 35–38] from living subjects, as these are the most easily available samples. If there is any information on chromium determination in tissues and organs, it comes rather from earlier publications [36, 39–44]; one of the newest was written in 2010 by Goullé et al. [45]. That is why, in this study, an evaluation of total chromium content in the internal organs and blood of non-exposed and non-poisoned subjects from Southern Poland was carried out.

## Material and Methods

### Reagents and Instrumentation

Analytical grade reagents from Merck (Darmstadt, Germany) and deionized water (NANOpure Diamond, Barnstead, Dubuque, IA) were used in the analysis. Glass and polypropylene vessels were soaked for 24 h in 5% (*v*/*v*) nitric acid solution and rinsed before use with deionized water.

An Ethos 1 microwave digestion system (Milestone, Sorisole, Italy) equipped with Teflon high-pressure reaction vessels was used for the wet digestion of samples of investigated materials.

The determination of total chromium was performed by an electrothermal atomic absorption spectrometer (Solaar MQZe, Thermo Electron, Waltham, MA), with Zeeman background correction, at a wavelength of 357.9 nm (slit width of 0.7 nm), according to a previously optimized and validated four-step procedure [46]. The measured volume of the sample solution was 20 μL.

### Material and Sample Preparation

The study was carried out on 60 autopsy cases performed routinely in the Department of Forensic Medicine of the Jagiellonian University Medical College in Kraków, after approval by the Bioethics Committee of the Jagiellonian University (reference number: KBET/102/B/2009). Biological material undergoing testing was taken from the deceased not environmentally and occupationally exposed to elevated levels of chromium after visual assessment by a forensic medical examiner. Sections of internal organs (weighing about 50–100 g) were removed with stainless steel scalpels, put into acid pre-washed polypropylene vials, and frozen at − 20 °C until analysis. Before the digestion procedure, the samples were partially thawed, and thick sections of the surrounding surface tissue, which could have been contaminated by dust and/or stainless steel material at an earlier stage, were cut off using an acid pre-washed plastic knife (similarly to the procedure described by Rahil-Khazen et al. [42]). Then the whole sample was homogenized, and approximately 1.5 mL of blood and 1.5 g of internal organs was subjected to digestion: samples of biological material were wet digested with nitric acid and hydrogen peroxide in a 5:1 volume ratio in a microwave system. A total of 414 samples of postmortem blood and sections of macroscopically normal internal organs (brain, stomach, liver, kidneys, heart, and lungs) obtained from 21 women and 39 men, aged 29–89 (56 ± 18) years and 24–88 (47 ± 13) years, respectively, were analyzed.

The determination of chromium was performed on two parallel samples of one kind of biological material (taken from one deceased person)—giving a total number of 828 analyzed samples. Each analytical result was calculated as the average of the two values determined for a pair of samples (for a single sample, chromium concentration was measured in triplicate). Values of chromium concentrations found in blood and internal organs are presented in Table 1.

**Table 1:** Chromium concentration in blood and internal organs (number of samples, mean ± SD, median, range) in non-exposed population of Southern Poland [ng/g wet weight or ng/mL]—gender approach

Material	Group	*n*	Mean ± SD	Median	Range*	
Blood	Female	19	4.51 ± 3.53	4.33	0.11	11.4
	Male	37	4.67 ± 4.28	3.74	0.11	16.4
	Total	56	4.62 ± 4.01	3.85	0.11	16.4
Brain	Female	19	56.3 ± 53.3	39.5	4.8	197
	Male	36	34.3 ± 23.2	26.8	4.7	95.9
	Total	55	40.8 ± 33.4	29.6	4.7	136
Stomach	Female	19	92.0 ± 88.3	44.7	6.1	313
	Male	35	31.8 ± 16.4	27.7	11.6	76.4
	Total	47	32.6 ± 16.7	28.3	6.1	76.4
Liver	Female	19	136 ± 81	123	23	334
	Male	37	290 ± 367	162	11	1381
	Total	52	156 ± 124	122	11	506
Kidney	Female	17	50.1 ± 21.0	52.9	19.8	82.3
	Male	37	106 ± 92	68.5	2.9	371
	Total	54	85.2 ± 73.4	60.6	2.9	298
Lung	Female	19	362 ± 338	285	44	1204
	Male	32	271 ± 181	211	13	716
	Total	49	271 ± 189	207	13	798
Heart	Female	21	103 ± 104	59.2	3.6	366
	Male	37	84.5 ± 58.2	77.0	6.9	318
	Total	58	90.5 ± 74.9	70.4	3.6	320

*Minimum and maximum values obtained (after the removal of the outliers identified by the Grubb’s test) among different group analyzed (female, male, total)

### Statistical Analysis

The material undergoing testing was taken from individuals without any visible pathological changes in their bodies. However, during the study, in some samples of certain materials, chromium content was found to be extremely high in comparison to the mean value established for the whole group. Using Grubbs’ test for outliers, all these extreme results, possibly related to health, nutrition, or smoking habits (data to which we unfortunately did not have access), were rejected before statistical evaluation of the obtained data.

**Fig. 1:**
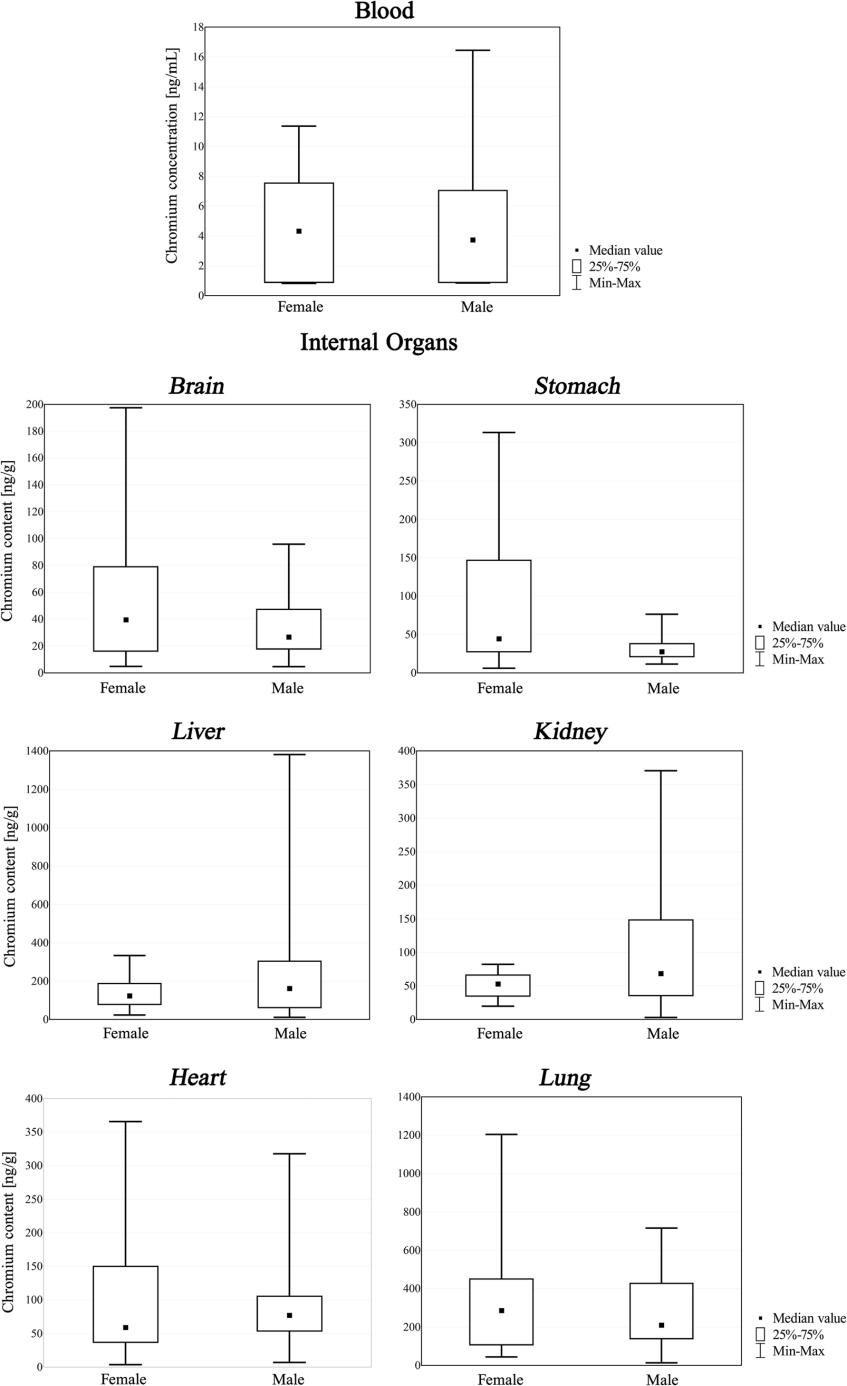
Chromium content in blood and internal organs [ng/mL or ng/g] of non-exposed population of Southern Poland: the Mann-Whitney *U* Test’s box and whisker plots drawn for gender

**Fig. 2:**
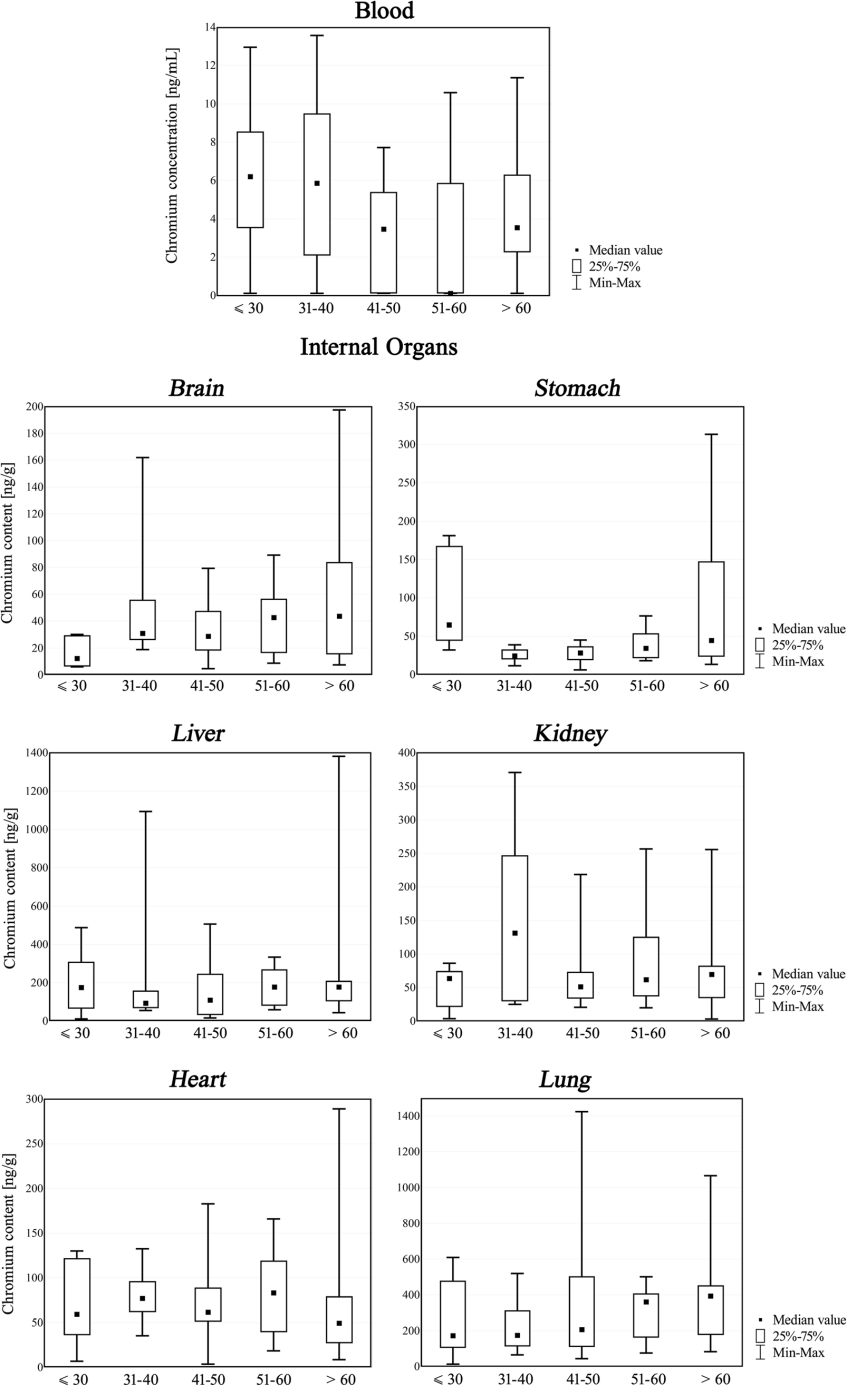
Chromium content in blood and internal organs [ng/mL or ng/g] of non-exposed population of Southern Poland: ANOVA Kruskal-Wallis Test’s box and whisker plots drawn for age-groups

During statistical examination, Statistica 5.0 software was used. The Mann-Whitney *U* Test and ANOVA Kruskal-Wallis Test were applied to assess the relationship between chromium content in postmortem material and gender or age—the box and whisker plots are presented in Figs. 1 and 2.

## Results and Discussion

In the analyses of body tissues, the highest chromium concentrations were determined in cumulative organs, such as the lungs and liver, in the ranges of 13.4–798 and 11–506 ng/g, respectively. The lowest chromium content was obtained for the stomach and brain, where the total averages did not exceed 50 ng/g. What is more, in 15 blood samples, the concentration of chromium was below the limit of quantification (LOQ, 0.22 ng/mL). For these samples, the results were replaced by a value of half the LOQ, when calculating the approximate mean chromium content.

Using the Mann-Whitney *U* Test, a statistically significant relationship between gender and chromium content (Table 1) was only revealed in the stomach (*p* = 0.006). It may be associated with different diets consumed by these two groups, as it is generally accepted that men eat more meat and bread (rich in chromium), while women consume more fruit, vegetables, and dairy products (with less chromium content) [3, 47–49].

As one can find in available literature, with the exception of the lungs, the concentration of chromium in all other organs decreases with age [6]. However in our study, such tendency could be seen only in blood samples; in other tissues, there was more or less visible increase in chromium content. What is more, when considering heart tissue, median values of chromium concentration obtained during the course of this study were practically on one level (between 50 and 80 ng/g) in all age groups, as well as in kidney tissue (close to 70 ng/g, apart from the group between 31 and 40 years where the value was about two times greater). ANOVA Kruskal-Wallis Test was used to assess whether there was a possible relationship between age and chromium concentration in particular tissue (Table 2). A statistically significant differences were again revealed only in the stomach samples (*p* = 0.01).

**Table 2:** Chromium concentration in blood and internal organs (number of samples, mean ± SD, median, range) in non-exposed population of Southern Poland [ng/g wet weight or ng/mL]—age-group approach

Material	Age-group	*n*	Mean ± SD	Median	Range*	
Blood	< 30	8	7.08 ± 3.41	7.58	0.11	12.9
	31–40	10	5.85 ± 4.37	5.85	0.11	13.6
	41–50	14	3.95 ± 2.35	3.89	0.11	7.71
	51–60	11	3.35 ± 3.87	1.96	0.11	10.6
	> 60	12	4.40 ± 3.63	3.54	0.11	11.4
Brain	< 30	6	18.2 ± 11.0	12.6	6.1	30.3
	31–40	9	57.2 ± 53.3	31.1	18.9	162
	41–50	17	37.7 ± 20.5	36.5	4.7	79.4
	51–60	11	45.4 ± 25.9	45.2	8.8	89.3
	> 60	12	59.7 ± 58.7	43.7	7.5	197
Stomach	< 30	7	87.2 ± 61.0	64.8	32.1	181
	31–40	9	25.4 ± 9.2	24.4	11.6	38.9
	41–50	15	27.0 ± 11.3	28.3	6.1	45.2
	51–60	10	39.2 ± 20.1	34.4	18.1	76.4
	> 60	13	93.0 ± 93.4	44.7	13.3	313
Liver	< 30	8	227 ± 162	219	10.9	488
	31–40	9	289 ± 403	93	54.8	1094
	41–50	18	177 ± 153	130	15.8	506
	51–60	8	199 ± 99	193	58.6	334
	> 60	13	317 ± 434	178	43.4	1381
Kidney	< 30	7	61.2 ± 22.5	66.0	21.1	86.2
	31–40	10	149 ± 121	131	24.9	371
	41–50	18	78.7 ± 60.4	55.3	20.4	218
	51–60	10	97.0 ± 73.1	68.5	19.8	257
	> 60	10	90.9 ± 87.4	69.6	2.9	256
Lung	< 30	8	267 ± 233	173	13.4	610
	31–40	8	223 ± 152	175	65.2	519
	41–50	15	427 ± 488	207	43.9	1424
	51–60	9	301 ± 153	367	75.2	501
	> 60	13	407 ± 297	393	83.0	1066
Heart	< 30	7	71.9 ± 45.6	59.2	6.89	130
	31–40	9	80.2 ± 31.9	77.0	35.4	133
	41–50	16	75.0 ± 48.8	61.6	3.63	183
	51–60	10	82.0 ± 48.7	83.2	18.5	166
	> 60	13	84.7 ± 90.9	49.4	8.64	289

*Minimum and maximum values obtained (after the removal of the outliers identified by the Grubb’s test) among different group analyzed (female, male, total)

In Tables 3 and 4, levels of chromium in blood and internal organs determined by different authors are given.

**Table 3:** Reference values of chromium in blood (number of samples, mean ± SD, range) found by various authors [ng/mL]

Group	*n*	Mean ± SD	Median	Range		Country	Reference
Male and female	23	0.18 ± 0.10				Denmark	[12]
Male and female	134	0.19		0.10	0.60	UK	[15]
Male and female	519	0.23 ± 0,01		0.09	0.75	Italy	[11]
Adolescents	200	0.25^c^		0.23^b^	0.27^b^	Belgium	[22]
Male and female	110	0.44 ± 0.27	0.38	0.12	1.07	Italy	[16]
Male and female	90	1.07 ± 0.68^a^	0.96^a^	0.04^a^	2.37^a^	Pakistan	[23]
School children	49	1.25 ± 0.74	1.10	0.40	4.63	South Africa	[18]
Male and female	106	4.10	0.55	0.33^b^	0.87^b^	France	[21]
Male and female	75	75.3 ± 7.6		63.3	87.1	Pakistan	[17]
Male 46–60 years	43	60.5 ± 3.2	61.3	58.1	63.8	Pakistan	[19]
Male 62–75 years	37	58.9 ± 2.6	58.4	55.2	61.1	Pakistan	[19]
Female 46–60 years	47	59.2 ± 2.9	59.1	57.1	63.5	Pakistan	[19]
Female 61–75 years	39	54.2 ± 2.8	54.4	51.9	57.9	Pakistan	[19]
Boys 6–10 years	42	91.6 ± 8.2		83.9	109.3	Pakistan	[20]
Girls 6–10 years	38	86.1 ± 7.2		79	93.2	Pakistan	[20]

^a^The conversion of g to mL has been made taking into account average density of blood—1.06 g/mL

^b^Range as 5–95th percentile

^c^Geometric mean

**Table 4:** Reference values of chromium in internal organs (number of samples, median, mean ± SD, range) found by various authors [ng/g]

Material	*n*	Mean ± SD	Median	Range		Country	Reference
Brain	22	81	84	37	120	Norway	[42]
	142	170 ± 120		150^a^	190^a^	Korea	[43]
	20	51		20	140	France	[45]
Liver	30		< 60	< 60	1800	Romania	[39]
	41	100	80	40^b^	210^b^	Italy	[36]
	148	330 ± 250		290^a^	370^a^	Korea	[43]
	5–11	270 ± 280				Sweden	[44]
	20	60		40	100	France	[45]
Kidney	30	–	< 30	< 30	120	Romania	[39]
	41	60	60	30^b^	120^b^	Italy	[36]
	28^c^	53	49	32	94	Norway	[42]
	28^d^	50	52	21	94	Norway	[42]
	139	180 ± 140		150^a^	200^a^	Korea	[43]
	5–11	513 ± 267				Sweden	[44]
	19	43		40	110	France	[45]
Lung	23		570	140	2190	Germany	[41]
	41	770		70^b^	1490^b^	Italy	[36]
	140	410 ± 320		350	460	Korea	[43]
	20	< LOQ				France	[45]
	18	61	57	41	103	Norway	[42]
Heart	140	170 ± 130		150	190	Korea	[43]
	20	< LOQ				France	[45]

^a^95% confidence interval

^b^Range as 5–95th percentile

^c^Kidney cortex

^d^Kidney medulla

When comparing results obtained in the course of this study with data published earlier, it can be stated that mean values of chromium concentration in blood and internal organs generally fell within the range of reference concentrations established by other authors, with a few exceptions. In blood samples, values obtained in normal adults given by Afridi et al. [17] and Kazi et al. [19] as well as in children (6–10 years old) reported by Shah et al. [20]—all from Pakistan—were definitely higher than those obtained by us and Cesbron et al. (among the French population) [21]. There have been no data concerning chromium content in the stomach provided by other authors. In the analysis of liver samples, chromium content in the range of 11–506 ng/g, with an average value of 156 ng/g, determined during this study was slightly higher than reported by Caroli et al. [36] and Goullé et al. [45] for non-occupationally exposed Italian and French subjects, respectively. Both Muramatsu and Parr [39] and Rahil-Khazen et al. [42] were not able to determine chromium in this medium. Among the results for the kidneys (in the range of 2.9–298 ng/g, with an average value of 85.2 ng/g) and the brain (in the range of 4.7–136 ng/g, with an average value of 40.8 ng/g), there were some higher ones; e.g., Engström et al. [44] estimated 513 ng/g in liver biopsy tissue supplied by Le Centre de Toxicologie du Québec (Canada) and Rahil-Khazen et al. [42]—81 ng/g in brain front lobe from autopsies performed in the Gade Institute, Department of Pathology and Department of Forensic Medicine (Norway). It is worth mentioning that compared to Yoo et al. [43], mean values of chromium concentration determined in this study were lower in all investigated matrices.

As mentioned in the Introduction section, one should bear in mind that the diversity between results obtained by us and other authors may be connected with the fact that different populations were studied in which many factors (such as health, age, gender, nutrition, and living and working environment) could have influenced the amount and distribution of chromium in the body. What is more, data obtained in this study, as well as the above mentioned results provided by other authors, refer to non-occupationally exposed, healthy people. Chromium content in blood and organs may vary in different pathologies and be higher when exposure to elevated levels occurs. For example, total chromium concentration ranges in the blood of atherosclerosis patients versus healthy donors, reported by Ilyas and Shah [23] were slightly higher (140–4210 and 40–2240 ng/g, respectively), while those in the blood of diabetes mellitus patients, as presented by Kazi et al., were lower (51.9–63.8 ng/mL in control group and 39.8–50.3 ng/mL in patients). When considering occupational exposure data provided by Danadevi et al. [50], Teraoka [51] are good examples. The first authors estimated that welders had significantly higher total chromium concentrations in blood when compared with controls (151.65 versus 17.86 ng/mL). The second reported that the highest concentration of chromium, from ten to a thousand times greater than the content stated for the control group (average 1.4 mg/g, dry weight), were found in lungs of two chromium plating (220 and 1400 mg/g) and three chromate refining (40, 58, and 110 mg/g) workers. A slightly higher values were also noticed in other investigated material, e.g., the liver, heart, spleen, and kidney.

## Conclusions

On the basis of the results of analyses of chromium content in postmortem material obtained from 60 people in a Southern Polish population, it can be stated that there were no significant differences (except for the stomach) between male and female subjects, as well as that obtained data were generally consistent with other published findings, when taking into consideration that many factors may influence the final results. The obtained data may constitute a contribution to population-based studies on metal content in biological material, in particular autopsy material, and may be useful in the interpretation of the results of chemo-toxicological investigations.
